# A Novel One-Stop Multidisciplinary Clinic for Chronic Postoperative Inguinal Pain: Initial Experiences and Outcomes

**DOI:** 10.3389/jaws.2025.14317

**Published:** 2025-06-16

**Authors:** Federica Cocciolo, Alice Birch, Maciej Pawlak, Lucy Miller, Alan Bennett, Matthew Lund, David Sanders, John M. Findlay

**Affiliations:** ^1^ Academic Department of Abdominal Wall and Upper Gastrointestinal Surgery, North Devon District Hospital, Royal Devon University Healthcare NHS Foundation Trust, Barnstaple, United Kingdom; ^2^ Department of Abdominal Wall Surgery, Golden Jubilee National University Hospital, Clydebank, United Kingdom; ^3^ Department of Anaesthetics and Pain Management, North Devon District Hospital, Royal Devon University Healthcare NHS Foundation Trust, Barnstaple, United Kingdom; ^4^ Department of Clinical and Biomedical Sciences, University of Exeter Medical School, University of Exeter, St Luke’s Campus, Exeter, United Kingdom; ^5^ NIHR Exeter Biomedical Research Centre, University of Exeter Medical School, University of Exeter, St Luke’s Campus, Exeter, United Kingdom

**Keywords:** chronic postoperative inguinal pain (CPIP), multidisciplinary clinic, patient-reported outcomes (PROMS), hernia surgery complications, pain management

## Abstract

**Background:**

Chronic Postoperative Inguinal Pain (CPIP) affects 10%–20% of patients following inguinal hernia repair, persisting for over 3 months post-surgery. It involves a complex interplay of neuropathic and nociceptive pain, secondary sensitization, and functional and psychological impacts. The condition often coexists with other pain causes, complicating diagnosis and treatment. Despite recommendations for multidisciplinary management, diagnostic and treatment pathways are frequently fragmented.

**Objective:**

This study evaluated the efficacy of a one-stop multidisciplinary clinic for CPIP in improving patient-reported outcomes (PROMS) and satisfaction.

**Methods:**

A one-stop multidisciplinary clinic was established at the North Devon Comprehensive Hernia Centre, involving an Abdominal Wall Surgeon, Advanced Clinical Practitioners, Pain Management Consultants, and Pain Specialist Physiotherapists. Following a remote ACP assessment, patients underwent 45-minute evaluations by a surgeon, pain specialist, and physiotherapist, culminating in an MDT discussion and a personalized management plan. Data were retrospectively collected for patients reviewed between July 2021 and July 2022, including demographics, surgical history, CPIP diagnoses, treatments, and PROMS.

**Results:**

Forty patients underwent MDT assessment; 55% pursued further treatment. Among 19 patients with follow-up data, 26% underwent surgery, 35% invasive non-surgical treatments, and 39% pharmacological therapies combined with physiotherapy and psychological support. Pain scores (VAS) decreased from 7.2 to 2.8, and functional activity (mAAS) improved from 20.3 to 9.7 (p < 0.0001). Patient satisfaction was high (mean score: 4.5/5).

**Conclusion:**

The one-stop multidisciplinary clinic significantly improved pain, function, and satisfaction, highlighting its value for CPIP management. Larger studies with delayed follow-up are needed to validate these findings.

## Introduction

Chronic Postoperative Inguinal Pain (CPIP) is a common and important condition affecting approximately 10%–20% of patients who undergo inguinal hernia repair [1–4]. CPIP is defined as persistent pain in the groin area lasting more than 3 months after surgery [4], and can represent a complex interplay of neuropathic and nociceptive pain, with secondary sensitisation, and functional and psychological consequences [5, 6]. CPIP can also coexist with other causes of persistent and acute pain [7].

As such, it can be extremely challenging to assess, diagnose, investigate and treat. Whilst some recommendations have been developed to help clinicians navigate this complexity, in particular advocating a multidisciplinary approach [4, 8], anecdotally patients’ diagnostic and treatment pathways tend to follow a sequence of separate, isolated episodes (for example, a surgical consultation to exclude hernia recurrence or mesh abnormality, proceeding to reoperation, or referral to a separate chronic pain clinic if no target is found [9, 10].

However, the complexity of CPIP suggests that there is a need for a combined and synchronous model of multidisciplinary care, which may be of benefit both clinically and holistically, which can assess and address all aspect of CPIP in conjunction with each other [1, 5]. The aim of this study was to develop a new, dedicated, one-stop multidisciplinary specialist clinic for CPIP, and evaluate the efficacy of this service in terms of patient reported outcomes (PROMS) and satisfaction.

### Establishing a One-Stop Multidisciplinary Clinic

To address this need, we established a novel one-stop multidisciplinary clinic at the North Devon Comprehensive Hernia Centre, North Devon District Hospital, which provides a tertiary referral service for complex abdominal wall surgery and CPIP. This clinic comprises a team of a Consultant Abdominal Wall Surgeon with a specialist interest in CPIP, Advanced Clinical Practitioners (ACP) in abdominal wall surgery, two Consultants in Pain Management, and two Pain Specialist Physiotherapists.

The goal of the clinic is to provide a comprehensive, joined-up diagnostic and therapeutic service for patients, and a bespoke management plan designed in collaboration with them, and centred around their circumstances, goals and concerns, generated in real time.

### Process

The initial referral is screened by a Consultant Abdominal Wall Surgeon with a specialist interest in CPIP, who confirms the clinic is an appropriate environment for assessment and management on the basis of the referral documents, and contacts the referrer or patient if neccessary. Patients receive an initial remote assessment by an ACP, who conducts a comprehensive patient consultation about their CPIP history (including their symptoms and pain before their initial hernia surgery), how this impacts upon their physical and psychological wellbeing, previous operations and interventions, other related medical and pain history, and their goals, expectations and concerns, and collates information regarding any relevant investigations and procedures (for example, the operation note from any initial hernia repair) [4]. This is recorded using a standardised template on our Electronic Health Record, and allows any outstanding or necessary information of investigations to be retrieved or performed in advance of their clinic appointment, and provides a framework for the MDT clinic [11].

Patients then attend for their CPIP appointment, at which they undergo triple 45 min assessment by a surgeon, Pain Specialist and Pain Specialist Physiotherapist. First, they undergo assessment by a Consultant Abdominal Wall Surgeon in conjunction with the ACP, who reviews the above aspects, and performs a detailed physical examination (hernia, soft tissue and orthopaedic) including standardised pain and sensation mapping, and reviews any imaging [8, 9]. Whilst this is an holistic assessment, there is a particular focus on determining the underlying cause(s) of CPIP, and identifying any particular diagnostic or therapeutic targets. These include evidence of hernia recurrence, mesh or fixation-related abnormalities (such as meshoma), identifying tender points for local anaesthetic and steroid injection, or evidence of nerve involvement.

Patients then undergo a specialist pain assessment with a Consultant in Pain Management, with a particular focus on the nature and cause(s) of the pain, any pharmacological options for further management. Following discussion with the surgeon, they then perform any targeted diagnostic or therapeutic ultrasound-guided tender point and nerve blocks with local anaesthetic +/- corticosteroid. As corticosteroid can risk a flare of pain this is carefully considered on the basis of whether it is plausible that there is active inflammation which may be attenuated by the addition of steroid (such as inflammation from a meshoma or suture), or whether the intent is purely diagnostic.

Patients then receive a Pain Specialist Physiotherapy assessment, with a particular focus on assessing for any co-existant musculoskeletal pathology (such as sciatica, hip arthritis, or tendinitis), assessing and addressing any secondary musculoskeletal effects (such as guarding or stiffness), and any psychological effects. This also provides an opportunity to assess for any effects of an injection or nerve block performed immediately before.

Whether the physiotherapy assessment is undertaken before or after any injection therapy is tailored to the patient. Most commonly it is performed afterwards; if local anaesthetic is completely or partly successful, then the physiotherapist is able to quantify its degree of efficacy at rest and during provoking movements, and its impact upon function. However, coincident musculoskeletal and psychological factors can still be assessed; such as secondary guarding, and adjacent hip or back pain which is not affected by any nerve block.

After triple assessment a MDT discussion aims to offer patients a diagnosis and cause(s) of their CPIP (for example, neuropathy of a specific nerve, nociception, superficial scar-related allodynia, hernia recurrence, secondary sensitisation) within the context of any other relevant pain, medical and surgical history; a holistic understanding of any secondary effects this is having (such as guarding, anxiety, sexual or physical dysfunction); and a bespoke management plan, centred around their individual goals and expectations. Management may involve targeted nerve therapies (repeat steroid injection, radiofrequency ablation, or surgical neurectomy), targeted nociceptive therapies (repeat steroid injection, mesh or suture explantation), repair of any hernia recurrence, general physiotherapy and rehabilitation, pain-specific physiotherapy, cognitive therapies, and systemic or topical pharmacological therapies. Where possible, the MDT recommends interventions in parallel, and the next step depending on the results of investigations or interventions. Patients are reviewed routinely to determine whether treatment has been effective, however, the time point at which they are re-reviewed depends on the length of treatment recommended and whether a subsequent treatment had been suggested. As this recommendation is derived from a dynamic discussion of complimentary disciplines consensus is reached without significant differences of opinion.

## Methods

### Study Design

This was a retrospective cohort analysis of all patients reviewed in the MDT CPIP clinic at between July 2021 and July 2022 during its first year. The study was registered as a service evaluation with the Trust’s Clinical Governance Department and confirmed to be exempt from ethical approval using the UK HRA Decision tool.^1^

### Data Collection

Data were collected retrospectively from patient medical records (paper and electronic record (EPIC, Epic Systems, Verona, Wisconsin, USA). These comprised data routinely collected before and after the CPIP clinic including patient basic demographics (age and sex), surgical and treatment history, CPIP diagnoses and management plans, and patient reported outcome measures (PROMS): pain (Visual Analogue Scale; (VAS from 0-10), functional activity (modified Activity Assessment Scale; (mAAS; 0-40) [12] and satisfaction (assessed using a Likert scale 1-5. Follow-up data were collected 3 months post-intervention to assess changes in pain levels, functional activity, and patient satisfaction.

### Data Analysis

Mean VAS and mAAs were compared using paired t tests. P < 0.05 was adjusted for multiple comparisons using a Bonferonni correction, with p < 0.025 considered significant.

## Results

**FIGURE 1:**
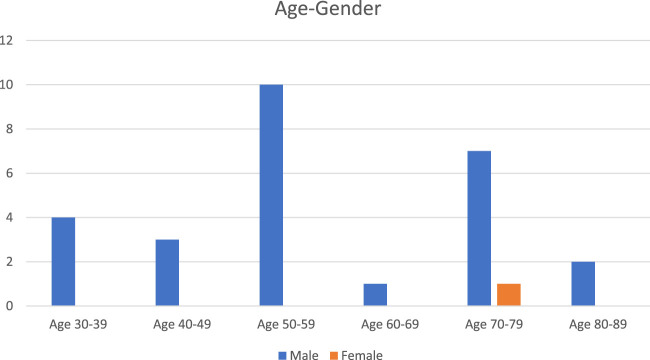
“Age and Gender Distribution: A comparison of male and female participants across various age groups”.

Forty patients underwent MDT assessment during the study period; 39 men and 1 woman (Figure 1). All received a bespoke treatment plan including advice regarding analgesics and activity. Twenty-two (55%) went on to further formal treatment (beyond management advice and general rehabilitation physiotherapy), with follow up data available for 19 (86.4%) patients (Table 1).

**TABLE 1:** Summary table of MDT CPIP study outcomes.

Category	Details		
Total Patients Assessed	40 (39 men, 1 woman)		
Further Formal Treatment Required	22 patients (55%)		
Follow-up Data Available	19 of 22 (86.4%)		

Diagnosis Types			
Category			
Musculoskeletal	Hip impingement, joint degeneration		
Neuropathic Pain	Sensitization component		
Surgical Causes	Meshoma, cord lipoma, nerve entrapment		

Follow-Up Treatment Among 19 Patients			
Treatment Type	No. of Patients		
Surgery	5	26% (e.g., meshoma removal, triple neurectomy)	
Invasive Non-Surgical (e.g., RFA, injections)	6	35%	
Physiotherapy & Psychological Support	7	39%	
Combination Therapies	5	- 3: Cognitive therapy + physiotherapy - 2: RFA + physio + cognitive	

Pain and Function Scores			
Metric	Pre-Treatment	Post-Treatment	Statistical Significance
VAS Score (Pain)	7.2 ± 1.9 (range 4–10)	2.8 ± 1.5	p < 0.0001
mAAS Score (Function)	20.3 ± 7.3	9.7 ± 5.2	p < 0.0001 (mean change: −10.6)

Patient Satisfaction			
Metric	Result		
Average Satisfaction Score	4.5 / 5		
Qualitative Feedback	Appreciated coordinated, individualized MDT care		

### Diagnosis and Treatment

Patients were diagnosed with either musculoskeletal (MSK) pathologies such as hip impingement, joint degeneration, or neuropathic pain with a sensitization component, or pain caused by meshoma, cord lipoma, or nerve entrapment.

Of those patients who underwent further treatment with follow-up data, 26% (5 patients) had surgery (specifically, removal of meshoma, triple neurectomy, laparoscopic TEP procedure with removal of previous mesh, and exploration of the groin with mesh explantation, triple neurectomy). Additionally, 35% (6 patients) received invasive non-surgical nerve directed treatment (radiofrequency ablation, with or without prior rounds of local anaesthetic +/- steroid injections under ultrasound guidance in 2 of these), while 39% (7 patients) underwent musculoskeletal or specific pain-management physiotherapy and psychological support. Beyond these combinations, 5 patients underwent multiple synchronous treatments, largely cognitive therapies with musculoskeletal physiotherapy (3 patients), but also interventional therapies (radiofrequency ablation) in combination with musculoskeletal physiotherapy and cognitive therapies (2 patients).

At the initial assessment, all patients reported moderate to significant pain, with a mean Visual Analog Scale (VAS) score of 7.2 ± 1.9, ranging from 4 to 10. Following the treatment, there was a significant reduction in pain, with the mean VAS score decreasing to 2.8 ± 1.5 post-intervention. To determine the statistical significance of this reduction, a paired t-test was conducted, which revealed a highly significant decrease in pain levels (p < 0.0001).

In terms of functional activity, the mean baseline Modified Activity Assessment Scale (mAAS) score was 20.3 ± 7.3. Post-intervention, this score improved to 9.7 ± 5.2, with a mean change of −10.6 ± 2.6. This substantial improvement indicates a significant enhancement in functional activity. The paired t-test analysis confirmed the significance of this improvement, yielding a p-value <0.0001. Specifically, patients reported enhanced mobility and a reduction in activity-related pain following the intervention.

### Satisfaction

Patient satisfaction with the MDT approach was high, with an average satisfaction score of 4.5 out of 5. Specifically, patients reported that they appreciated the comprehensive and coordinated care provided by the multidisciplinary team, highlighting the benefits of individualized treatment plans and the holistic approach to managing CPIP.

## Discussion

CPIP is common, often complex, and can be extremely challenging for patients and clinicians [3, 6]. CPIP differs from many surgical and persistent pain conditions in the range and coexistence of causes and treatment options, and these factors mandate a multidisciplinary approach [4, 5]. Whilst this is often advocated, anecdotally this is usually provided in discrete, separate consultations and episodes, rather than a combined, synchronous one-stop service with a MDT discussion [13]. We describe what we believe is the first one-stop MDT clinic for CPIP, with our initial outcomes.

We identified a range of often coexisting diagnoses, including musculoskeletal (MSK) pathologies such as hip impingement, joint degeneration, or neuropathic pain with a sensitization component, as well as pain caused by meshoma, cord lipoma, or nerve entrapment. In just over half of the patients, we identified additional treatment options, which included surgery, percutaneous radiofrequency ablation, nerve or tender point injections, and/or pharmacological therapy. For these patients, as well as those without such therapeutic options, we also initiated targeted or general rehabilitation physiotherapy and psychological interventions and advice. Overall, patients reported substantial and significant improvements in both their pain and function at 3 months, along with high levels of satisfaction with the service. Therefore, we conclude that this represents a valuable model of care for our patients, with the results of surgery in keeping with those described in the literature [1, 2], although the absence of a comparative cohort significantly limits the strength of these conclusions.

We believe there may be a number of reasons underlying the benefits seen with this clinic model. The high patient satisfaction scores may reflect the value of taking a MDT and patient-centred approach in managing a complex chronic pain condition. In particular, a holistic aspect is incorporated into every assessment and MDT discussion, and allows for a thorough evaluation of CPIP, considering physical, psychological, and social factors related to the pain, rather than merely concentrating on “nerves” or “inflammation,” or procedural targets. This comprehensive approach aims to ensure that all potential sources of pain are identified and addressed, and synchronous assessment and immediate MDT discussion ensures that each specialist is able to discuss their impressions within the context of their colleagues’, and discuss the relative risks and benefits of treatment options.

This ensures that each patient in the MDT clinic receives a personalized treatment plan tailored to their specific needs and circumstances. Whilst sadly sometimes we have not been able to identify a target for physical or pharmacological treatment, we are still able to offer psychological and often physical therapies. This individualized approach may enhance patient believe in and commitment to the treatment regimen and so improve outcomes. The combination of pharmacological management, surgical intervention, physical therapy, and psychological support addresses the multifaceted nature of CPIP, leading to more effective pain relief and functional improvement.

This high level of patient satisfaction with the MDT approach may also show the importance of patient-centred care in managing chronic pain conditions. By actively involving patients in their care and incorporating their feedback into treatment plans, the MDT clinic may foster a sense of empowerment and ownership among patients.

This study has a number of limitations. Whilst robust recording of routine clinical data was undertaken, as a retrospective analysis there may be bias introduced by data recording. There may also be measurement bias introduced by comparing PROMS undertaken in different settings (for example, in person and remotely), as well as patients not wishing to express any true dissatisfaction with the service. This analysis also lacks a control group to compare and generalise findings, and the different and non-standardised lengths of follow up might affect treatment efficacy, firstly due to allowing time for multiple interventions, and secondly by allowing more time for these interventions to work and for any pain that might naturally improve to do so. This patient group also represents a highly selected cohort (by virtue of their referral to this service).

There are a number of potential areas by which to refine this service. Firstly, at referral no formal quantitative or qualitative screening is undertaken (such as pain or symptom scores, or psychometric); this may be of benefit in organising clinics and tailoring care still further. However, we have since altered the referral screening process to include a multidisciplinary recommendation as to whether patients may in some circumstances not require all three extended consultations, and so improve access and efficiency.

## Conclusion

CPIP is common, complex and challenging to manage. We report a new, one-stop multidisciplinary assessment clinic, which has shown significant and substantial improvements in PROMS, both patient pain and function [7]. This will require more delayed follow up, and a larger study population to better determine its efficacy, as well as validation in another hospital setting [1, 3]. However, we believe this represents a valuable and novel way to provide high quality care to patients with CPIP.

## Data Availability

The original contributions presented in the study are included in the article/supplementary material, further inquiries can be directed to the corresponding author.

## Footnotes

1
https://www.hra-decisiontools.org.uk
